# Modulation by Endothelin-1 of Spontaneous Activity and Membrane Currents of Atrioventricular Node Myocytes from the Rabbit Heart

**DOI:** 10.1371/journal.pone.0033448

**Published:** 2012-03-29

**Authors:** Stéphanie C. Choisy, Hongwei Cheng, Godfrey L. Smith, Andrew F. James, Jules C. Hancox

**Affiliations:** 1 School of Physiology & Pharmacology, Medical Sciences Building, University of Bristol, Bristol, United Kingdom; 2 Cardiovascular Physiology, University of Glasgow, Glasgow, United Kingdom; Brigham & Women's Hospital - Harvard Medical School, United States of America

## Abstract

**Background:**

The atrioventricular node (AVN) is a key component of the cardiac pacemaker-conduction system. Although it is known that receptors for the peptide hormone endothelin-1 (ET-1) are expressed in the AVN, there is very little information available on the modulatory effects of ET-1 on AVN electrophysiology. This study characterises for the first time acute modulatory effects of ET-1 on AVN cellular electrophysiology.

**Methods:**

Electrophysiological experiments were conducted in which recordings were made from rabbit isolated AVN cells at 35–37°C using the whole-cell patch clamp recording technique.

**Results:**

Application of ET-1 (10 nM) to spontaneously active AVN cells led rapidly (within ∼13 s) to membrane potential hyperpolarisation and cessation of spontaneous action potentials (APs). This effect was prevented by pre-application of the ET_A_ receptor inhibitor BQ-123 (1 µM) and was not mimicked by the ET_B_ receptor agonist IRL-1620 (300 nM). In whole-cell voltage-clamp experiments, ET-1 partially inhibited L-type calcium current (I_Ca,L_) and rapid delayed rectifier K^+^ current (I_Kr_), whilst it transiently activated the hyperpolarisation-activated current (I_f_) at voltages negative to the pacemaking range, and activated an inwardly rectifying current that was inhibited by both tertiapin-Q (300 nM) and Ba^2+^ ions (2 mM); each of these effects was sensitive to ET_A_ receptor inhibition. In cells exposed to tertiapin-Q, ET-1 application did not produce membrane potential hyperpolarisation or immediate cessation of spontaneous activity; instead, there was a progressive decline in AP amplitude and depolarisation of maximum diastolic potential.

**Conclusions:**

Acutely applied ET-1 exerts a direct modulatory effect on AVN cell electrophysiology. The dominant effect of ET-1 in this study was activation of a tertiapin-Q sensitive inwardly rectifying K^+^ current via ET_A_ receptors, which led rapidly to cell quiescence.

## Introduction

The atrioventricular node (AVN) is a small yet critically important component of the cardiac pacemaker-conduction system that lies at the junction between right atrium and ventricle [1], [2]. It is normally the only site where electrical activity can pass from atria to ventricles [1], [2]. Comparatively slow conduction through the AVN co-ordinates the normal timing of atrial then ventricular excitation [2], [3] and in the setting of supraventricular tachycardias such as atrial fibrillation, this limits impulse transmission to the ventricles [2], [4]. On the other hand, aberrant AVN conduction can itself lead to arrhythmia [2], [5]. The AVN also has pacemaking properties [2], [5]. Normally these are subordinate to the heart's dominant pacemaker the sinoatrial node (SAN); however, should the SAN fail the AVN can take over pacemaking of the ventricles [2], [5]. AVN pacemaking is incompletely understood, but is established to involve an interplay between the activity of a number of different ionic conductances [6]–[10].

Endothelin-1 (ET-1) is a potent vasoactive peptide hormone that is produced constitutively within the heart by vascular and endocardial endothelial cells. There is also evidence for ET-1 release by cardiac myocytes [11], [12]. In addition to its vasoconstrictor action, endogenous release of the hormone is known to modulate the inotropic state of the heart and is also suggested to play a role in modulation of the heart rate (e.g. [13]–[16]). Elevated production and release of ET-1 is strongly implicated in the pathogenesis of heart failure and the generation of arrhythmias (for reviews see [17]–[19]). There is also evidence that ET-1 can be pro-arrhythmic independent of coronary vasoconstriction [12], [19]. Data from patients with angina pectoris have shown left bundle-branch block to be associated with raised ET-1 levels, suggesting that the hormone may be involved in conduction abnormalities [20]. Consistent with an ability of ET-1 to exert a direct effect on the pacemaker-conduction system, experiments on cells isolated from the rabbit SAN have demonstrated that ET-1 produces a negative chronotropic effect that is associated with direct ion channel modulation [21]–[23]. By contrast, to our knowledge, there is no current information available concerning direct effects of this peptide hormone on the AVN. Autoradiographic studies of the human myocardium have revealed a high density of ^125^I-ET-1 binding to the AVN and the penetrating and branching bundles of His, in addition to the atrial and ventricular myocardium [24]. Autoradiographic study of the porcine AVN has also demonstrated the presence of specific ^125^I-ET-1 binding sites in this region [25]. Indirect evidence that ET-1 can modulate AVN electrophysiology comes from electrocardiogram measurements from anaesthetised dogs and rats, which have shown that intra-coronary ET-1 administration can produce complete AV block [26], [27]. Although, when considered together, the effect of intra-coronary ET-1 and evidence for presence of ET receptors in the AVN are strongly suggestive of a direct action of ET-1 on AVN electrophysiology, they are not conclusive in this regard. The present study was therefore undertaken to address this gap in information, by using an established rabbit single AVN cell preparation [6], [28]–[32]. The study was conducted to establish the effects of ET-1 on: (i) AVN cell spontaneous activity and (ii) major ionic currents present in AVN. The results that emerge demonstrate that ET-1 exerts marked direct effects on AVN cell electrophysiology and also implicate endothelin-A (ET_A_) receptors in the observed modulatory actions.

## Materials and Methods

### Ethics statement

All procedures used in these experiments were approved by the University of Bristol ethics committee and adhere to the United Kingdom Home Office Animals Scientific Procedures Act of 1986.

### Rabbit AVN cell isolation

Male New-Zealand White rabbits (∼2.0 to 3.5 kg) were killed in accord with UK Home Office legislation, their hearts then rapidly excised and cells isolated from the entire atrioventricular nodal (AVN) region using an established enzymatic and mechanical dispersion technique [28], [29]; [33]. Isolated AVN cells were suspended in Kraft-Brühe “KB” solution [28], [34] and stored in a refrigerator until use.

### Electrophysiological recording

For recording, cells were transferred in KB solution into an experimental chamber (0.5 ml) mounted on the stage of an inverted microscope (Nikon Diaphot) and left to settle for 10 mins prior to superfusion with a normal Tyrode's solution, containing (in mM): NaCl 140, KCl 4, CaCl_2_ 2, MgCl_2_ 1, HEPES 5 and Glucose 10, (pH 7.4 with NaOH). ET-1 and other compounds were added to this solution. Patch-pipettes (Corning 7052 glass, AM Systems Inc, Sequim, WA, USA) were pulled using a P-97 Flaming/Brown micropipette puller (Sutter Instruments, Novato, CA, USA) and filled with a solution containing (in mM) [10]; [33]: KCl 110, NaCl 10, HEPES 10, MgCl_2_ 0.4, and Glucose 5, K_2_ATP dihydrate 5, GTP-Tris salt 0.5 (pH 7.1 with KOH). For ionic current but not action potential measurements the pipette solution also contained 5 mM BAPTA (*cf* [10], [33]). Recordings were made using an Axopatch 1D amplifier (Axon Instruments; now Molecular Devices, Sunnyvale, CA, USA). Pipette resistance was typically <3 MΩ; series resistance values were usually <7 MΩ (mean of 6.07±0.68 MΩ; n = 23) and ∼60–80% of the series resistance was compensated. Membrane capacitance values used for calculation of current densities (pA/pF) were obtained and compensated for using capacitance compensation on the recording amplifier; cell capacitance values obtained in this way have been shown previously to match closely those obtained using a ‘surge’ technique [28]. For voltage clamp experiments, membrane potential was held at −40 mV (as this corresponds to the zero current potential for rabbit AVN cells [28], [30]). Action potentials were recorded from spontaneously beating cells in current-clamp mode with zero-current injection, using a gap-free acquisition mode. Protocols were generated and data recorded using Clampex 8 (Axon Instruments; now Molecular Devices Sunnyvale, CA, USA). Data digitization rates were 10–25 kHz with an appropriate bandwidth of 2–10 kHz set on the amplifier.

### Solutions and Chemicals

Cells were superfused with experimental solutions at 35–37°C (checked regularly using a hand-held thermocouple). ET-1 and other compounds were applied externally to the cell under study using a home-built, rapid solution exchange device capable of exchanging superfusate in <1 s [35]. ET-1 (Sigma-Aldrich Company Ltd, Dorset, UK) was used at 10 nM from a stock solution of 100 µM prepared in 0.1% acetic acid. This concentration falls within the range of ET-1 concentrations used in prior cardiomyocyte studies (1–100 nM; e.g. [21], [23], [36]). The selective ET_A_ receptor antagonist BQ-123 (Sigma-Aldrich Company Ltd, Dorset, UK) was used at a maximally effective concentration of 1 µM [37] from a 1 mM stock solution made in deionised water, whilst the endothelin-B receptor (ET_B_) selective agonist IRL-1620 (Tocris Bioscience, Bristol, UK) [38] was superfused at a concentration of 300 nM from a 1 mM stock solution prepared in 0.1% acetic acid. Tertiapin-Q (Tocris Bioscience, Bristol, UK) and barium ions (Ba^2+^, as BaCl_2_, (Sigma-Aldrich Company Ltd, Dorset, UK)) were prepared in deionised water and respectively used at 300 nM and 2 mM. All drugs were aliquoted and stored at −20°C, except BaCl_2_ solution, which was stored at 4°C.

### Data analysis

Data were analysed and graphical plots produced using Clampfit 10.2 software (Molecular Devices Sunnyvale, CA, USA), Microsoft Excel (2003), GraphPad Prism (v5; GraphPad Software Inc, La Jolla, CA, USA), IgorPro (v3.16B, Wavemetrics Inc, Portland, OR, USA) and SigmaPlot (v12; Systat Software Inc, Chicago, IL, USA). Data are presented as mean ± standard error of the mean (SEM). Statistical analysis was performed using Student's *t*-test or two-way ANOVA with Bonferroni or Tukey post-hoc tests, as appropriate. Values of ‘p’ less than 0.05 were taken as significant.

Current-voltage relations for L-type Ca current (I_Ca,L_) and rapid delayed rectifier current (I_Kr_) were fitted with following equations:
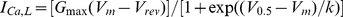
(1)where *G_max_* is maximal *I_Ca,L_* conductance, *V_m_* is the test potential at which *I_Ca,L_* was measured, *V_rev_* is the reversal potential determined from extrapolation of the ascending limb of plotted current-voltage relations, *V_0.5_* is the potential at which *I_Ca,L_* activation is half maximal and *k* is the slope factor describing current activation.
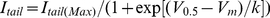
(2)where *I_tail_* represents I_Kr_ tail current amplitude recorded at −40 mV following a given test pulse membrane potential (*V_m_*) and *V_0.5_* and *k* have similar meanings to those for equation 1.

## Results

### Effects of ET-1 application on spontaneous APs

**Figure 1:**
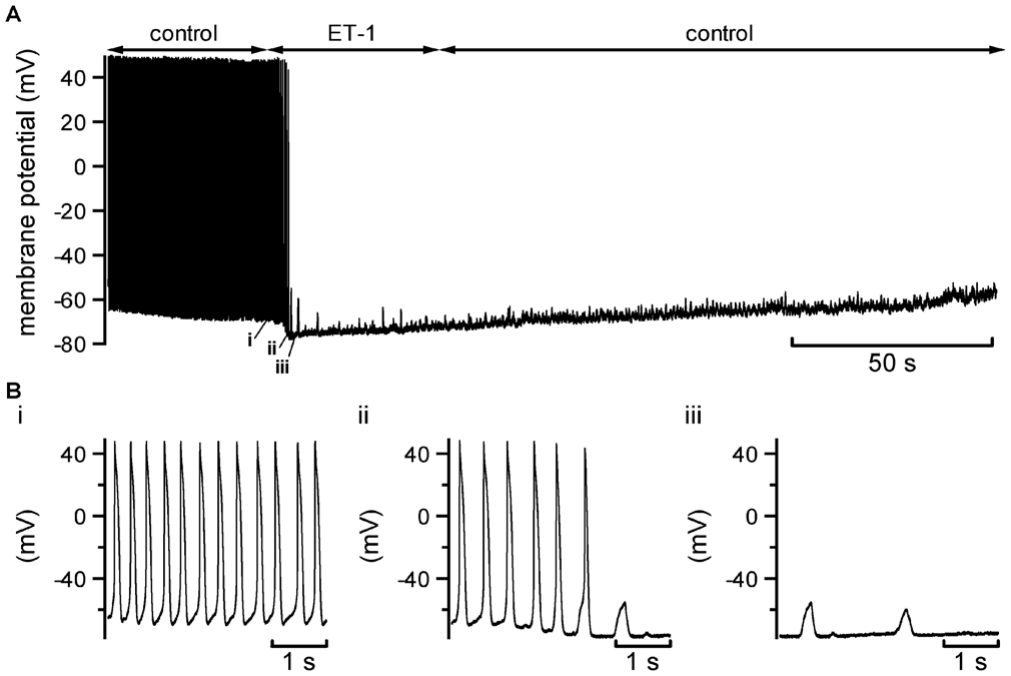
Effects of ET-1 on spontaneous APs. A. Slow time-base recording of APs before, during and after rapid application of 10 nM ET-1. B. Expanded (faster time-base) portions of the recording extracted from numbered sections of panel A (indicated labels i, ii, iii). Similar results were observed in 9 experiments.

Spontaneous APs were measured in whole-cell membrane potential recording mode from cells selected on the basis of exhibiting regular spontaneous activity (evidenced visually as regular spontaneous cell beating) during superfusion of control Tyrode's solution. Figure 1A shows a representative slow time-base record of APs, before, during and following exposure to 10 nM ET-1, whilst Figures 1Bi–iii show faster time-base extracts at the time-points indicated on Figure 1A. The mean spontaneous AP rate in control solution was 3.47±0.35 AP s^−1^ (n = 9), compatible with rates seen in previous studies [10], [28], [31], [33]). Application of ET-1 led rapidly to membrane potential hyperpolarisation and to cessation of spontaneous APs (within 13.1±2.1 s of ET-1 application; n = 9). As shown in Figure 1A, after reaching an initial peak response (the mean peak membrane potential hyperpolarisation produced by ET-1 in comparison to control maximum diastolic potential values was −20.3±2.0 mV, n = 9), membrane potential showed gradual depolarisation but without a return of spontaneous activity. As has been reported in some other studies (e.g. [21], [39]), the effect of ET-1 was not reversible on washout and membrane potential continued to show modest membrane potential depolarisation throughout the remainder of the measurement period. In all cells, whilst on ET-1 application cells rapidly ceased to generate spontaneous APs, small membrane potential oscillations were visible (of mean amplitude 3.5±0.3 mV, n = 9 cells; more than 25 oscillations analysed per cell), with occasional oscillations exceeding 10 mV; see Figures 1A and 1Bii and iii).

### Effects of ET-1 on ionic currents at negative membrane potentials

**Figure 2:**
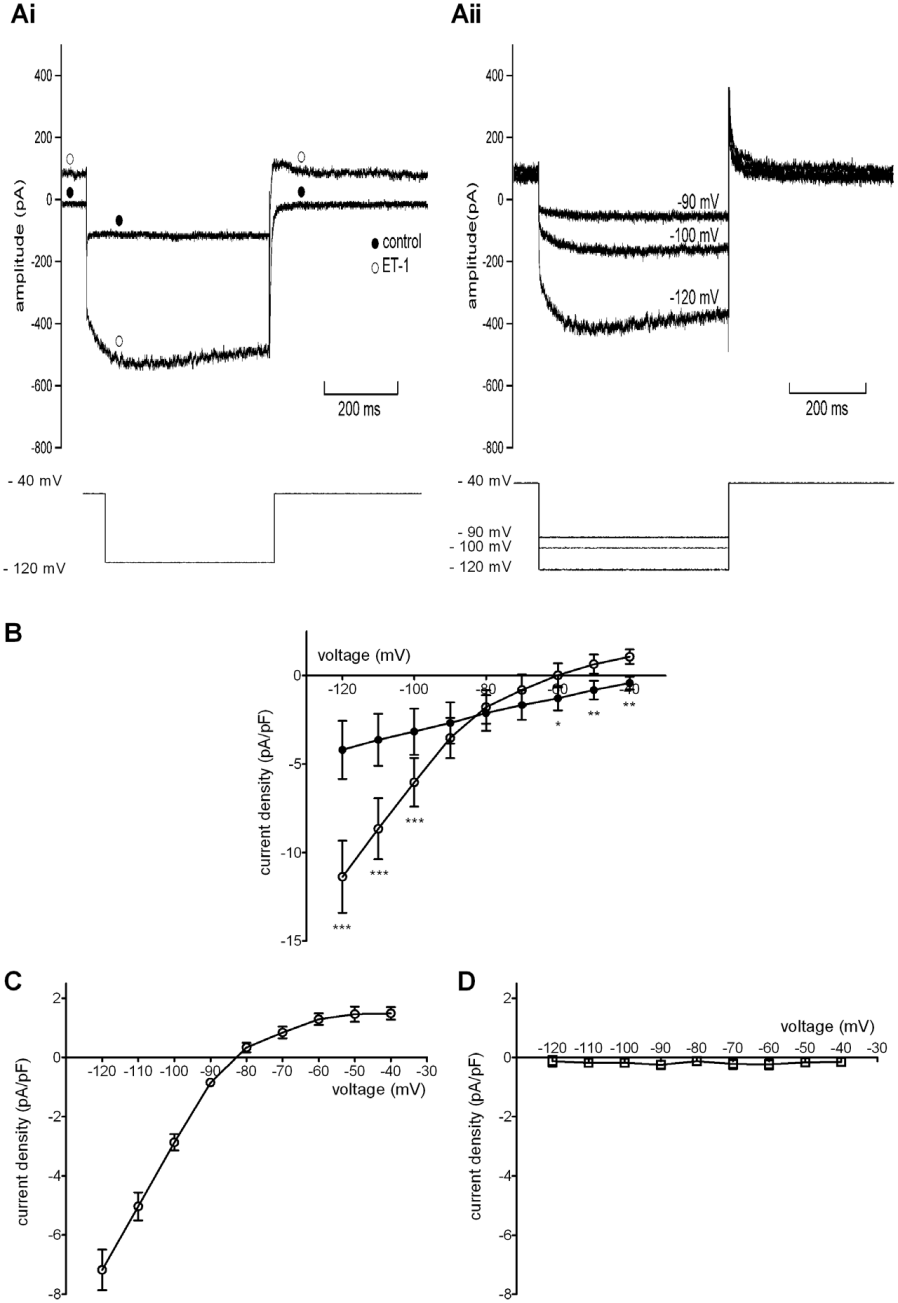
Modulation by ET-1 of instantaneous current in cells lacking I_f_. Ai. Currents recorded in the absence (control) and the presence of 10 nM ET at −120 mV (upper traces) when a voltage command was applied from −40 mV for 500 ms (lower trace). Note outward shift in holding current with ET-1. Closed circles indicate control trace; open circles indicate trace in ET-1. Aii. ET-1 activated currents (elicited at −90, −100 and −120 mV) obtained by digital subtraction of control from ET-1 records (same cell as Ai). B. Mean current-voltage (I–V) relationships for current measured at the start of applied voltage commands in absence (control, filled circles) and presence (open circles) of 10 nM ET-1 (n = 7). Asterisks denote statistical significance (p<0.05 *, p<0.01 **, p<0.001 ***). C. Plot of the mean I–V relationship for ET-1 sensitive difference (ET-1 activated) calculated from the same cells shown in B. D. Plot of ET-1 sensitive current when ET-1 was applied after 1 µM BQ-123 (n = 4).

AVN cell sub-types exhibiting time-independent and time-dependent current (I_f_) on membrane potential hyperpolarisation in standard extracellular solution have been identified [29], [31]. The effects of ET-1 were determined on cells with both response types. Effects of ET-1 were determined by application of 500 ms duration hyperpolarising voltage clamp commands: from a holding potential of −40 mV membrane potential was stepped to more negative potentials between −120 and −40 mV in 10 mV increments (at a pulse frequency of 0.2 Hz). Figure 2Ai shows current records elicited on membrane potential hyperpolarisation to −120 mV, for a cell that exhibited only time-independent current in control solution. On the application of ET-1, holding current shifted outwards (in control, holding current at −40 mV was −0.4±0.3 pA/pF, n = 7, NSD from zero current; in ET-1 this became 1.1±0.4 pA/pF; p<0.001 versus control). At −120 mV a large inward current was induced, with a marked increase in instantaneous inward current. Some time-dependence of the current can be seen in this example. Figure 2Aii shows ET-1 sensitive (activated) current from the same cell, at −90, −100 and −120 mV. Outward holding current is visible prior to the marked inward current elicited on membrane potential hyperpolarisation, the amplitude of which increased progressively with the magnitude of the applied hyperpolarising step. The stimulatory effect of ET-1 in augmenting time-independent (instantaneous) current was quantified by plotting current density-voltage relations for instantaneous current in control and in ET-1 (Figure 2B) and also by deriving from individual experiments the ET-1 sensitive difference (ET-1 activated) current, the mean of which is plotted in Figure 2C. Net instantaneous current (Figure 2B) was augmented by ET-1 across a range of potentials, with a left-ward shifted zero-current potential compared to control; control and ET-1 current-voltage relations intersected near −80 mV (Figure 2B). The ET-1 activated current (Figure 2C) showed marked inward rectification and reversed at ∼−82 mV (with a mean reversal potential derived from individual cell data of −82.1±1.2 mV; n = 7). The activation of this current accounts for the left-ward shift in zero current potential for net instantaneous current visible in Figure 2B. The ET-1 activated current was outwardly directed over potentials relevant to the diastolic potential range (typically between −65 and −40 mV). The ET-1 response showed some time-dependent ‘fade’ in the continued presence of the peptide: the mean amplitude of the ET-1 activated current at −120 mV immediately following (within 10–20 s) of ET-1 application was −7.2±0.7 pA/pF (n = 7), whilst at ∼2 minutes following ET-1 this had declined to −1.7±0.3 pA/pF (n = 7; p<0.001 versus immediate response) and in two cells that lasted ∼3.5 minutes following ET-1 this was −0.6±0.2 pA/pF. In cells pre-treated with BQ-123, ET-1 did not activate such a current: Figure 2D shows the instantaneous ET-1 sensitive difference current in cells exposed to BQ-123 prior to ET-1 application. This implicates ET_A_ receptor activation in the observed response.

**Figure 3:**
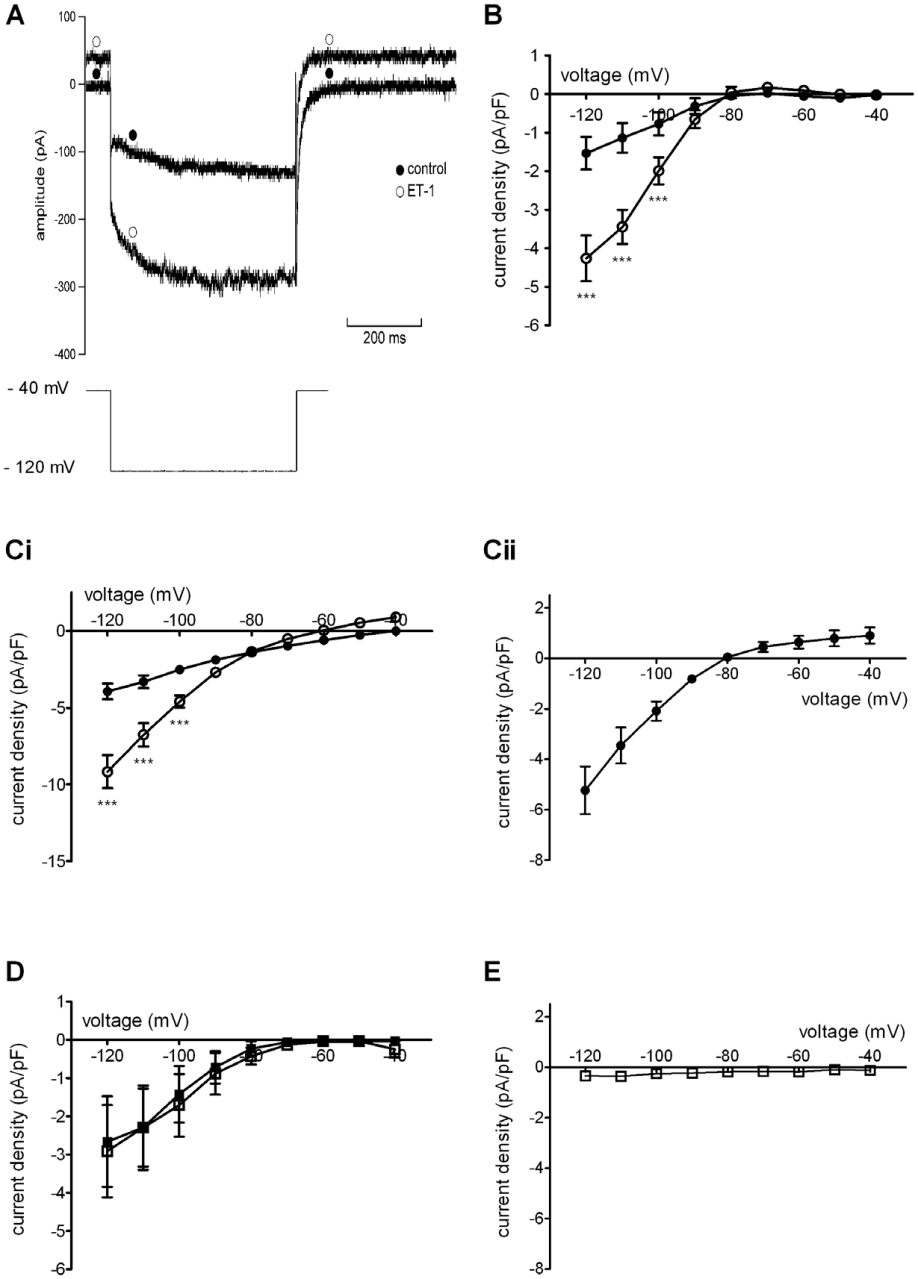
ET-1 effects on the hyperpolarisation-activated current I_f_. A. Upper traces show currents elicited −120 mV in an I_f_-expressing cell in control solution and 10 nM ET-1 by protocol shown in bottom trace. Note outward shift in holding current in presence of ET-1. Closed circles indicate control trace; open circles indicate trace in ET-1. B. Mean I–V relationships (n = 7) for I_f_, plotted as time-dependent current during command pulses, in absence (control, filled circles) and presence of 10 nM ET-1 (open circles). The activating effect of ET-1 was significant only at −120, −110 and −100 mV. C. Mean I–V relationships for the instantaneous current recorded at the beginning of the test-pulse (Ci: in absence (control, filled circles) and presence (open circles) of 10 nM ET-1). Cii shows I–V relation for the ET-1 activated instantaneous current (Cii, filled circles), in cells also showing I_f_ (n = 7). ET-1 activates a large inwardly rectifying current. D. I–V relations for I_f_ in presence of 1 µM BQ-123 (n = 11) without (filled squares) and with 10 nM ET-1 (open squares, n = 11 at all potentials except at −50 mV, where n = 10). BQ-123 prevented stimulation of I_f_ by ET-1. E. Inhibitory effect of 1 µM BQ-123 on the ET-1 activated current in cells exhibiting showing I_f_ (open squares, n = 12 except at −50 mV where n = 11). Asterisks denote statistical significance (p<0.001 ***).

Figure 3 shows comparable data for cells that exhibited I_f_ in control. Figure 3A shows representative currents in control solution and in the presence of ET-1. A dual effect was seen, in which both instantaneous and time-dependent current components were augmented in the presence of ET-1. Previously, I_f_ from AVN cells has been measured as the difference between end-pulse current and instantaneous current observed at the start of the hyperpolarising step [29], [33] and this method was used here to produce the mean I–V relations shown in Figure 3B. As reported previously [29], [33], under these conditions there was little I_f_ between −60 and −80 mV in control Tyrode's solution, whilst significant I_f_ was evident at more negative voltages. In the presence of ET-1, time-dependent current was significantly augmented at potentials of −100 mV and more negative than this (Figure 3B). However, this response was transient: the mean amplitude of I_f_ at −120 mV immediately following (within 10–20 s) of ET-1 was −4.3±0.6 pA/pF (n = 7), whilst at ∼2 minutes following ET-1 this was −1.9±0.7 pA/pF (n = 7; p<0.001 versus the immediate response), with the current at ∼3.5 minutes following ET-1 similar to that following 2 minutes of exposure. Figures 3Ci and Cii show effects of ET-1 on the instantaneous current component (i.e. comparable data to those shown in Figures 2B and 2C), demonstrating that activation of an inwardly rectifying instantaneous current also occurred in these cells. Figure 3Ci shows mean net instantaneous current in control and ET-1, again showing a left-ward shift in zero-current potential and intersection of the two relations close to −80 mV. Figure 3Cii shows the current-density plot for ET-1 activated current, which resembles that in Figure 2C. Note that the magnitude of the ET-1 activated current was not significantly different from that from cells that lacked I_f_, except at −120 mV (p<0.01 at this potential only). Figure 3D shows plots of time-dependent I_f_ in the presence of BQ-123 and BQ-123+ET-1. These were closely superimposed, implicating ET_A_ receptors in the effect of ET-1 on I_f_. Figure 3E shows that, similar to cells lacking I_f_ (Figure 2), ET-1 failed to activate instantaneous inwardly rectifying current in the presence of BQ-123.

### Effects of ET-1 on I_Ca,L_

**Figure 4:**
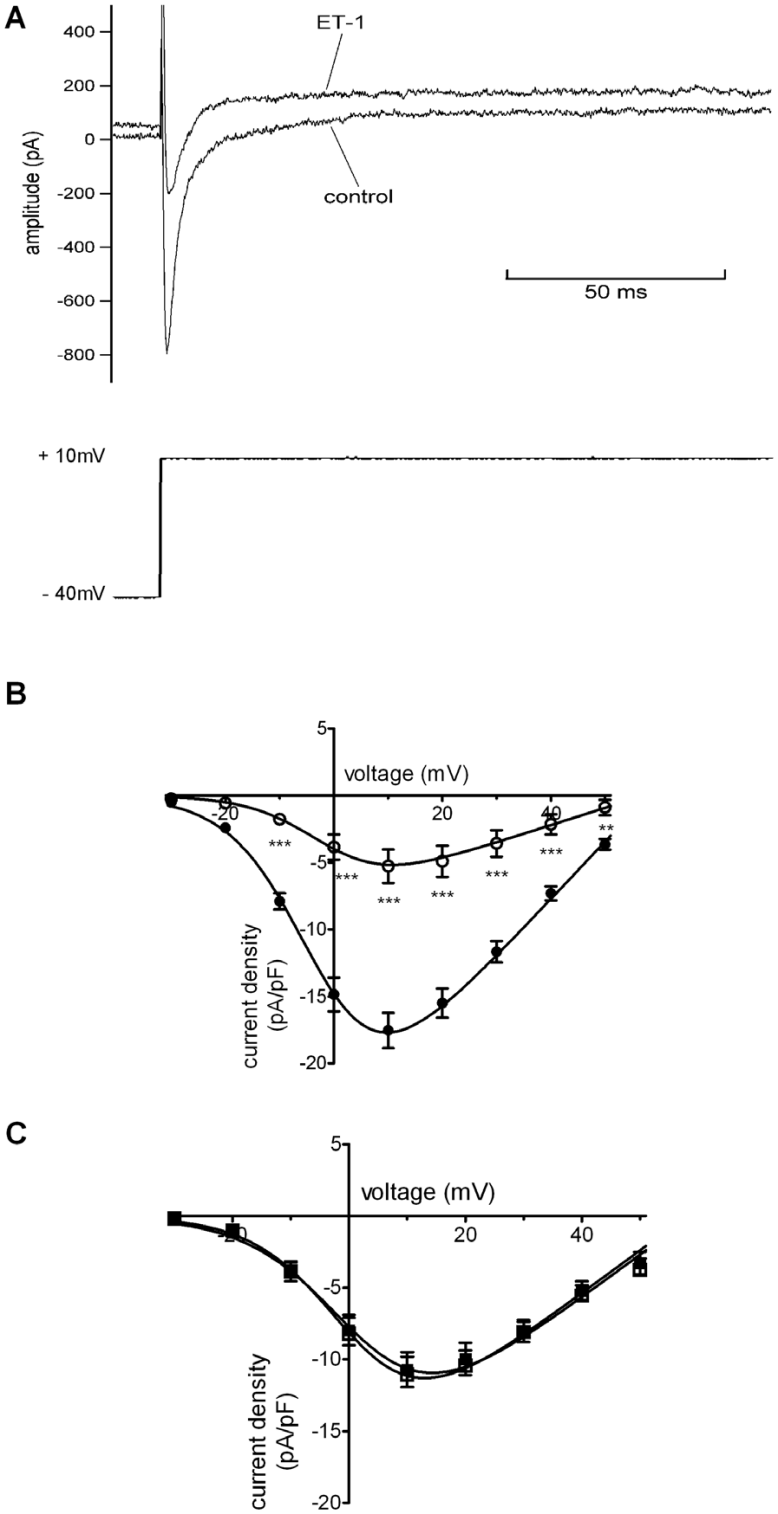
Effects of ET-1 on the L-type Ca^2+^ current (I_Ca,L_). A. Representative records of I_Ca,L_ (upper traces) elicited at +10 mV from a holding potential of −40 mV by protocol shown in lower traces, in both the absence (control) and presence of 10 nM ET-1. B. Plots of the mean I–V relations for I_Ca,L_ (n = 11), measured as peak - end pulse difference current, in control (filled circles) and with ET-1 (open circles): I–V curves were fitted using equation 1 (Methods) to give V_0.5_ values of −2.7±0.6 mV in control and −1.0±1.9 mV in ET-1 (p>0.1). Corresponding k values were 7.1±0.5 mV and 6.5±1.7 mV in control and ET-1, respectively (p>0.7). C. I–V relations (n = 12) for I_Ca,L_ in the presence of 1 µM BQ-123 (filled squares) and when 10 nM ET-1 was applied in the maintained presence of BQ-123 (open squares). V_0.5_ values were 2.1±1.1 mV and 1.0±1.0 mV respectively for BQ-123 and BQ-123+ET-1 (p>0.4), with corresponding k values of 8.2±0.9 mV and 7.4±0.9 mV (p>0.5). Asterisks in ‘B’ denote statistical significance (p<0.01 **, p<0.001 ***).

Similar to other recent studies from our laboratories [10], [33], the effects of ET-1 on I_Ca,L_ were determined from currents measured over a range of test potentials (500 ms commands were applied to voltages between −30 and +50 mV from a holding potential of −40 mV). Figure 4A shows representative traces of I_Ca,L_ elicited by depolarisation from −40 mV to +10 mV in the absence and presence of ET-1, showing a marked suppression of peak current amplitude by ET-1. Figure 4B shows mean current-voltage relations for I_Ca,L_ in control and with ET-1. Repeated applications of the I_Ca,L_ measurement protocol in the presence of ET-1 did not lead to significant further reductions in current beyond that shown in Figure 4A. A fit to the data with equation 1 yielded an activation V_0.5_ of −2.7±0.6 mV in control and of −1.0±1.9 mV in ET-1 (n = 11 cells; p>0.1), whilst G_max_ values derived from the fits to the data were 0.36±0.03 nS/pF (control) and 0.16±0.03 nS/pF (ET-1; p<0.001). In 12 further experiments, cells were exposed to the ET_A_ receptor antagonist BQ-123 (1 µM) prior to ET-1 application. Figure 4C shows mean I–V relations from these experiments: with BQ-123 application prior to ET-1 superfusion, ET-1 did not reduce I_Ca,L_ amplitude at any membrane voltage, implicating ET_A_ receptor activation in this action of ET-1. In 6 cells in which I_Ca,L_ at +20 mV was monitored during ET-1 exposure and following washout, the inhibitory effect of ET-1 was not reversed by washout.

### Effects of ET-1 on rapid delayed rectifier K^+^ current, I_Kr_

**Figure 5:**
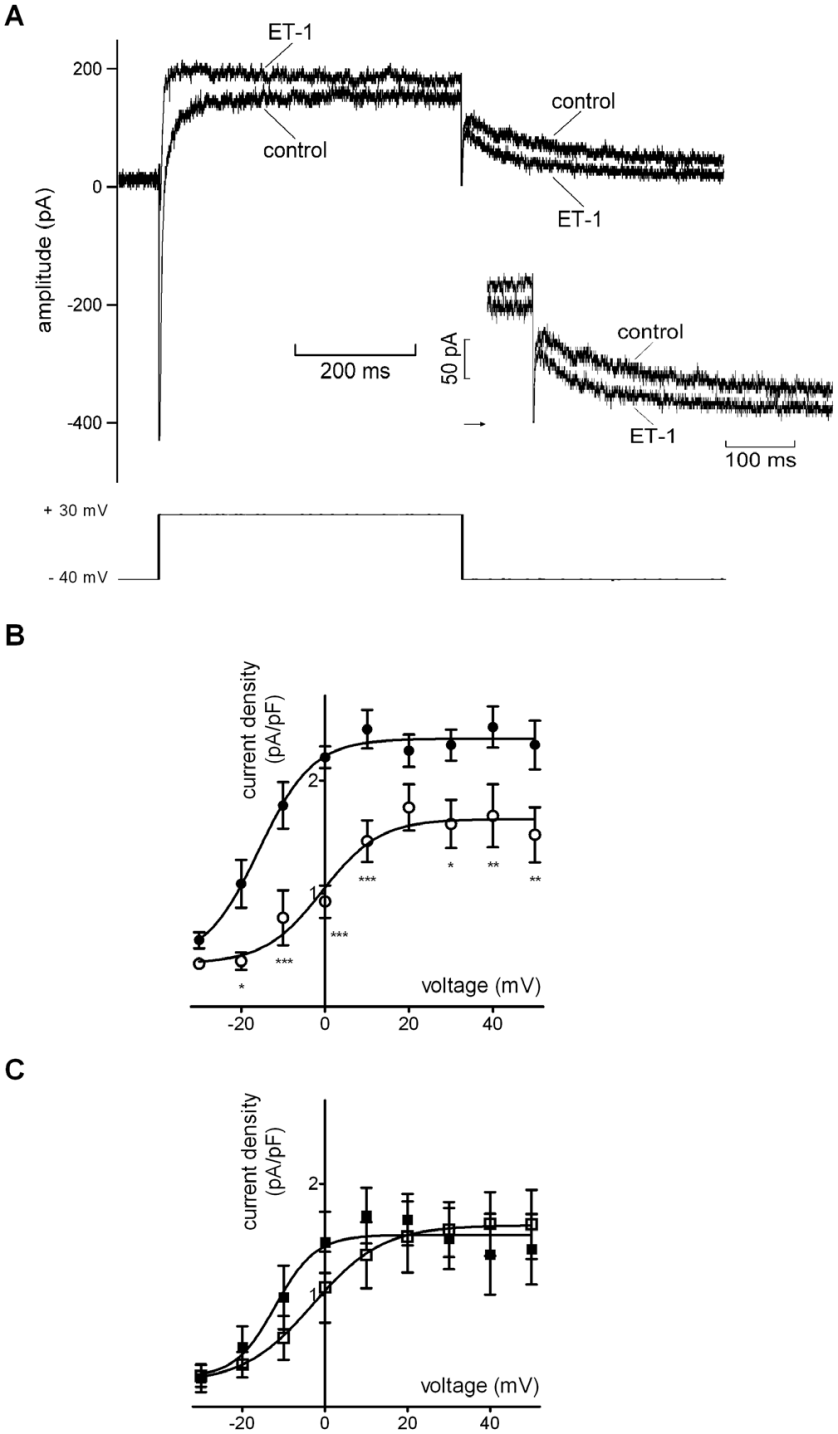
Effects of ET-1 on rapid delayed rectifier K^+^ current tails. A. Upper traces show currents elicited on depolarisation to +30 mV and subsequent repolarization to −40 mV by protocol shown in lower trace. Deactivating tail currents on repolarization represent the I_Kr_ ‘tail’. Currents are shown in control solution and in presence of 10 nM ET-1. Insert shows an expanded portion of the traces to highlight the ‘tail’ currents (the horizontal arrow in the inset denotes the zero current level). B. Mean ‘tail’ current I–V relationships for 5 cells, in absence (control, filled circles) and presence (open circles) of 10 nM ET-1. I–V curves were fitted with equation 2 (Methods) to derive V_0.5_ values of −15.7±3.6 mV in control and −0.7±4.3 mV in ET-1 (p<0.05), with respective k values of 6.4±2.6 mV and 6.9±3.9 mV (p>0.9). The ‘tail’ current was significantly reduced in presence of ET-1 at all voltages ranging from −20 to +50 mV except +20 mV. C. Mean I–V plots for I_Kr_ tails in the presence of 1 µM BQ-123 without (filled squares; n = 5) and with 10 nM ET-1 (open squares, n = 5 for all, except at +40 and +50 mV where n = 4). Derived V_0.5_ values were −12.0±5.1 mV and −2.9±6.4 mV for BQ-123 and BQ-123+ET-1, respectively (P>0.3), with associated k values of 4.9±4.4 and 8.4±5.8 (p>0.6). Asterisks in B denote statistical significance (p<0.05 *, p<0.01 **, p<0.001 ***).

The rapid delayed rectifier K^+^ current, I_Kr_, is important to AVN AP repolarisation and can also influence spontaneous rate [33], [40]–[42]. I_Kr_ is typically measured from AVN cells as outward tail current on repolarisation to a negative voltage following depolarising voltage commands, and lacks contamination from potentially overlapping currents such as I_Ks_, which is absent from rabbit AVN cells [33], [41]–[44]. Accordingly, the effects of ET-1 on I_Kr_ were assessed by measurements of outward tail current amplitude at −40 mV, following 500 ms voltage commands to more positive potentials (between −30 and +50 mV). Figure 5A shows representative traces elicited following depolarisation to +30 mV in the absence and presence of ET-1, with the inset displaying the tails currents on a higher gain. ET-1 produced a 20–30% decrease in tail current amplitude. Figure 5B shows mean I–V relations for I_Kr_ tails in control and at steady-state in ET-1 (n = 5). Tail currents were significantly reduced at nearly all potentials between −20 and +50 mV. A fit to the data with equation 2 yielded activation V_0.5_ values of −15.7±3.6 and −0.7±4.3 mV in control and ET-1 respectively (p<0.05; n = 5). Figure 5C shows mean tail current I–V relations for five experiments on cells exposed to BQ-123 prior to ET-1. There was no significant difference between BQ-123 alone and ET-1 in the presence of BQ-123 in tail current at any test voltage, implicating ET_A_ receptor activation in mediating the suppressive effect of ET-1 on I_Kr_ tail amplitude. Although ET-1 in the presence of BQ-123 still appeared to produce some right-ward shift in the fit to the current-voltage relationship (V_0.5_ of −12.0±5.1 mV in BQ-123 alone and −2.9±6.4 mV in BQ-123 and ET-1; n = 5), due to cell-to-cell variability in response the difference did not attain statistical significance (p>0.3). Similar to I_Ca,L_ the effects of ET-1 on I_Kr_ did not reverse on ET-1 washout.

### Investigation of the mechanism of AP quiescence

**Figure 6:**
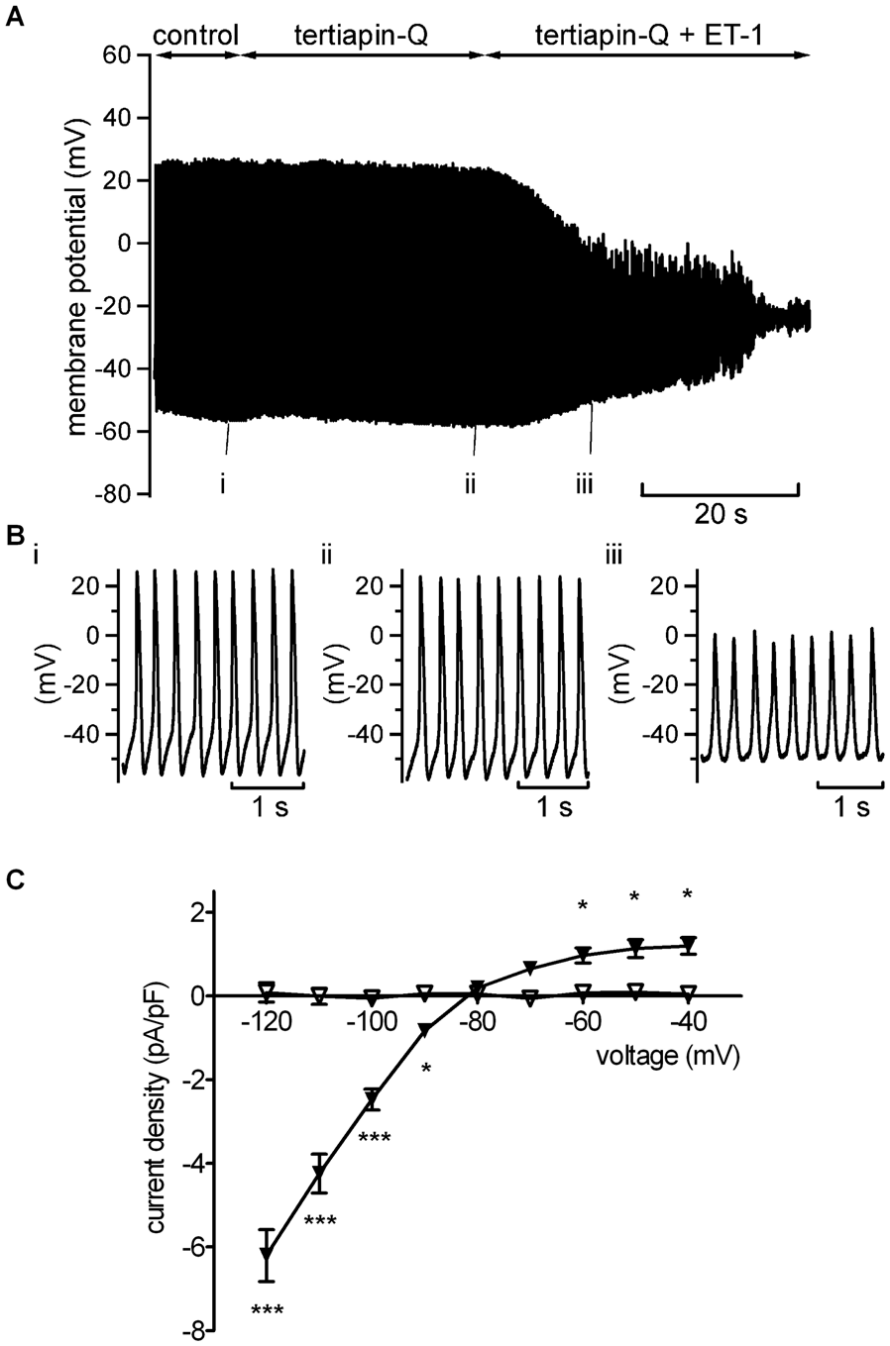
Effects of tertiapin-Q (TQ) on the effect of ET-1 on spontaneous APs and ET-1 activated current. A. Continuous recording of spontaneous activity in control, in the presence of TQ (300 nM) before and with application of 10 nM ET-1 in the maintained presence of TQ. Note the absence of immediate hyperpolarisation and cessation of APs evident in Figure 1. Bi, ii and iii show expanded records from recording in A, at time-points indicated: i taken during control, ii near the end of TQ alone and iii is taken at ∼13 seconds of ET-1 application (at which time-point cells exposed to ET-1 alone had hyperpolarised and become quiescent). Similar results were obtained from 7 cells. C. Mean I–V relationships for the 10 nM ET-1 activated instantaneous current in absence (filled triangles, n = 14) and in presence of 300 nM TQ (open triangles, n = 7. except at −80 mV where n = 6). TQ prevented this action of ET-1. Asterisks in C denote statistical significance (p<0.05 *, p<0.001 ***).

The rapid membrane potential hyperpolarisation with ET-1 during spontaneous AP recording shown in Figure 1 is consistent with the activation of an outward current over the diastolic membrane potential range. The ET-1 activated current observed here (Figures 2 and 3) exhibited a voltage dependence reminiscent of inwardly rectifying K^+^ current in AVN cells activated by acetylcholine (I_K,ACh_) or adenosine (I_K,Ado_), each of which exert marked negative chronotropic effects on isolated AVN cells (e.g. [28], [30]). Tertiapin/tertiapin-Q have been shown previously to inhibit sinoatrial I_K,ACh_ (e.g. [45], [46]) and to inhibit atrio-ventricular block induced in the guinea-pig by I_K,ACh_ activation [47]. We hypothesised, therefore, that if ET-1 activates a current similar to I_K,ACh_ in AVN cells, tertiapin-Q should prevent rapid ET-1 induced membrane potential hyperpolarisation and hyperpolarisation-associated cell quiescence. Consequently, experiments were performed in which tertiapin-Q was applied prior to ET-1 superfusion during spontaneous AP recording. Figure 6A shows representative results. Application of 300 nM tertiapin-Q alone did not alter spontaneous activity; however when ET-1 was subsequently applied in the maintained presence of tertiapin-Q, no rapid MDP hyperpolarisation or hyperpolarisation-associated quiescence was induced. Instead, there was a gradual depolarisation of MDP and decrease in AP amplitude (this cell depolarised and became quiescent within ∼30 seconds of ET-1 in the presence of tertiapin-Q). Similar experiments were performed on seven spontaneously active cells; in none of them did ET-1 induce membrane potential hypolarisation in the presence of tertiapin-Q. Experiments were then performed to determine whether or not tertiapin-Q also inhibited ET-1 activated current under voltage clamp. Figure 6C shows that, in the presence of tertiapin-Q, ET-1 was unable to activate any inwardly rectifying instantaneous current. In a further 7 experiments, a second known inhibitor of inwardly rectifying ACh-activated K^+^ conductances, Ba^2+^ ions, was applied (at 2 mM; [48]) during repetitive application of a descending voltage-ramp protocol. Ba^2+^ rapidly inhibited the ET-1 activated current in all cells tested (not shown). Collectively, the results of these experiments implicate ET-1 activation of an I_K,ACh_-like current in the rapid suppression of spontaneous activity shown in Figure 1.

**Figure 7:**
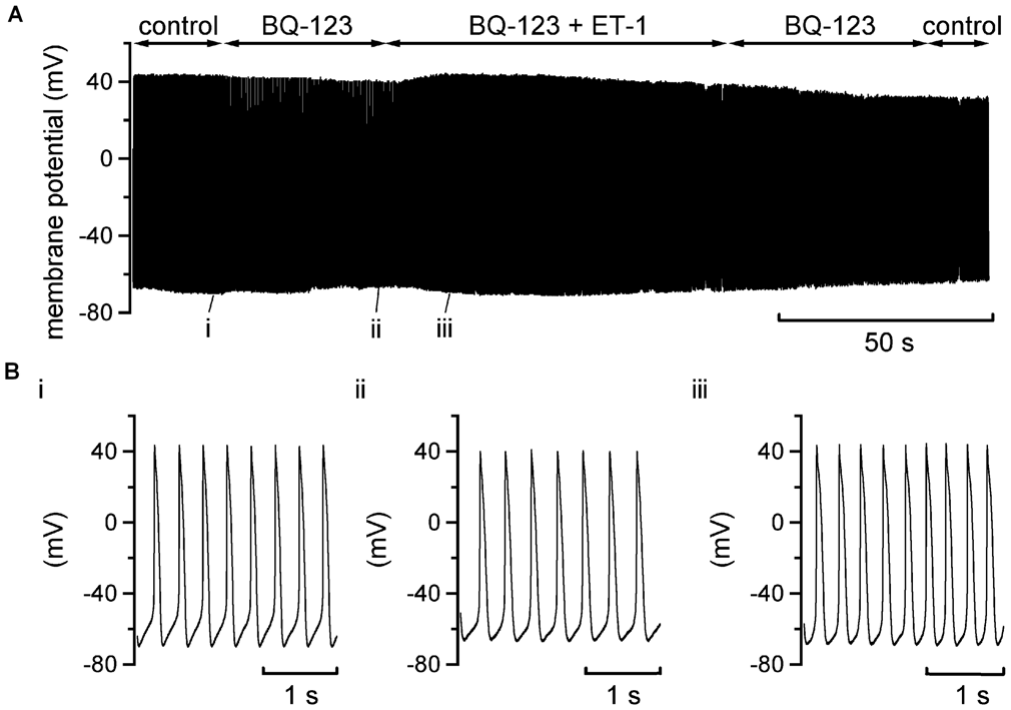
BQ-123 inhibition of the effects of ET-1 on spontaneous APs. A. Continuous recording of spontaneous activity in control solution, in presence of 1 µM BQ-123 before and during subsequent superfusion of 10 nM ET-1 in the maintained presence of BQ-123. BQ-123 prevented the ET-1 induced cessation of spontaneous APs and associated membrane potential hyperpolarisation. Bi, ii and iii show expanded extracts of the recording in A, at the time-points indicated: i taken during control, ii near the end of BQ-123 alone and iii is taken at ∼13 seconds of ET-1 application in the presence of BQ-123 (at which time-point cells exposed to ET-1 alone had hyperpolarised and become quiescent). Similar results were obtained in 6 cells.

**Figure 8:**
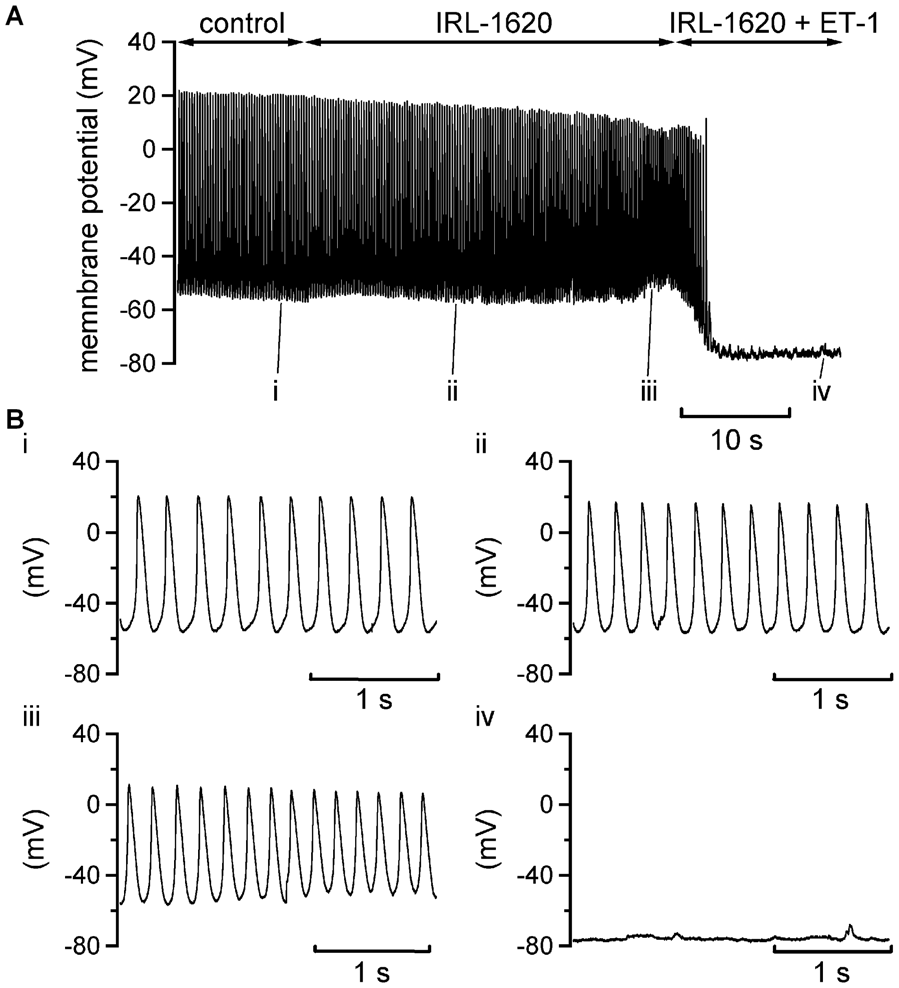
Effect of IRL-1620 on spontaneous APs. A. Slow time-base continuous recording of APs in control conditions, followed by the application of 300 nM IRL-1620 and following addition of 10 nM ET-1 in the maintained presence of IRL-1620. IRL-1620 did not abolish the spontaneous APs, whereas application of 10 nM ET-1 stopped spontaneous activity and hyperpolarized the membrane potential. Similar results were acquired in 5 cells. Bi, ii, iii and iv show expanded extracts of the recording shown in A, at the time-points indicated: i taken during control, ii and iii taken respectively mid-way and towards the end of IRL-1620 alone and iv is taken at ∼13 seconds of ET-1 application in the presence of IRL-1620 (at which time-point cells exposed to ET-1 alone had hyperpolarised and become quiescent).

ET-1 effects on all currents observed under voltage-clamp were sensitive to ET_A_ receptor inhibition (Figures 2, 3, 4, and 5). Therefore, additional spontaneous AP recordings were performed in which the ET_A_ receptor inhibitor BQ-123 was applied prior to ET-1 superfusion. Figure 7 shows the results from one of six similar experiments. When ET-1 was applied following exposure of cells to BQ-123 no rapid membrane potential hyperpolarisation or suppression of spontaneous activity was observed. In a final set of experiments, the effects of a selective ET_B_ receptor agonist, IRL-1620 were investigated. IRL-1620 produced a small depolarisation of MDP (by 5.2±1.4 mV; n = 5) and decrease in AP overshoot (a mean reduction of 14.1±1.4 mV; see Figure 8); however, no cell tested responded to IRL-1620 with membrane potential hyperpolarisation or quiescence. In contrast, when ET-1 was applied in the maintained presence of IRL-1620 it produced rapid membrane potential hyperpolarisation and quiescence (similar to that seen when ET-1 alone was applied; Figure 1).

## Discussion

### Placing novel findings in context

To our knowledge, the present data are the first to demonstrate directly modulation by ET-1 of AVN electrophysiology. Previously, intravenous administration of BQ-123 to anaesthetised pigs and, via intracoronary injection, to a small sample of human patients with coronary artery disease has been reported not to alter AV nodal conduction [49], [50], suggestive of a lack of effect of basal endogeneous ET-1 on AV nodal conduction *via* ET_A_ receptors in those studies. On the other hand, intracoronary bolus injection of ET-1 to anesthetized dogs [26], [27] and rats [26], [27] has been shown to produce AV block, which is suggestive of an ability of increased ET-1 levels to modulate AVN electrical behaviour. The results of the present study show that ET-1 can suppress AVN cell activity, and the modulatory effects on AVN cellular electrophysiology seen here may help explain prior observations of ET-1 induced AV block [26], [27]. This is also the first study to demonstrate tertiapin-Q sensitivity of an ET-1 activated K^+^ current in any cell or tissue type. Several features of our findings merit more detailed discussion.

### Comparison with previous studies

Whilst the effects of ET-1 on cardiac ion channel currents from other cardiac cell types have been widely studied, the most relevant data for comparison with the present study come from prior investigations of isolated *sinoatrial* node (SAN) cell and tissue preparations [21]–[23]. In 2001 Ono and colleagues demonstrated negative chronotropic effects of ET-1 on rabbit intact SAN preparations and isolated SAN cells [23]. Application of ET-1 at the same concentration used in the present study (10 nM) was able to induce quiescence in isolated SAN cells, which either did not readily reverse or reversed incompletely during the recording measurement period. However, the negative chronotropic effect of ET-1 seen in that study was accompanied by membrane potential hyperpolarisation only in some (rod-shaped) cells, whilst other (spindle-shaped) cells showed depolarisation of the MDP [23]. A separate study of rabbit SAN cells by Tanaka and colleagues produced results in good agreement with those in the present investigation: application of 10 nM ET-1 routinely led rapidly to cessation of spontaneous activity and to membrane potential hyperpolarisation [21]. In the present study, all spontaneously active AVN cells exposed to ET-1 exhibited rapid membrane potential hyperpolarisation and quiescence. Voltage-clamp data from both SAN studies indicated a marked inhibitory action of ET-1 on I_Ca,L_ (∼50% inhibition at 10 nM ET-1) [21], [23] which is in reasonable agreement with our own observations for AVN cells. This contrasts with more modest effects of ET-1 on guinea-pig ventricular I_Ca,L_ (∼21% inhibition of basal I_Ca,L_ by 20 nM ET-1 [51] and human ventricular I_Ca,L_ (∼33% inhibition of basal I_Ca,L_ by 8 nM ET-1 [52]). Such differences may be accounted for by regional and/or species differences in ET-1 response, with rabbit primary and secondary pacemaker cell types exhibiting a greater response than ventricular myocytes from these two other species. The possibility of regional differences in response is supported by results from a study of rabbit ventricular myocytes [36] that reported weak bi-phasic effects of 10 nM ET-1 on basal I_Ca,L_, with an inhibitory effect of smaller magnitude (∼20%) than those seen for AVN (this study) or SAN [21], [23]. Ono and colleagues also reported a modest reduction in delayed rectifier (I_K_) tails across a wide range of voltages for spindle-shaped cells and up for voltages up to ∼0 mV for rod-shaped cells, accompanied in both SAN types by positively shifted (∼+5–+14 mV) I_K_ activation [23]. Tanaka and colleagues also showed suppression of SAN I_K_ by ET-1, though they did not study the voltage-dependence of the effect [21]. The activation V_0.5_ values under control conditions for the tail currents investigated in this study are within the range reported previously for AVN I_Kr_ [33], [41]. Although, in contrast with rabbit AVN cells that exhibit only I_Kr_ [33], [41]–[44], both I_Kr_ and I_Ks_ have been seen in rabbit SAN cells [43], [53], the activation characteristics of the current sensitive to ET-1 in the study of Ono and colleagues [23] are concordant with a primary identity as I_Kr_. Both the present study and that of Ono and colleagues [23] report right-shifted current activation with ET-1, suggesting modification of a similar current in each case. Consequently, it is highly likely that, as reported previously for human ventricular I_Kr_ [52], ET-1 inhibits I_Kr_ from both secondary and primary pacemaker cell types.

Previously reported effects of ET-1 on I_f_ from SAN cells show some differences from those observed here for AVN myocytes. Thus, Ono and colleagues reported that 10 nM ET-1 activated I_f_ between ∼−75 and −85 mV, but that it inhibited I_f_ at more negative voltages [23]; Tanaka and colleagues also reported an inhibitory action [22]. On the other hand, we saw significant augmentation of AVN cell I_f_ by ET-1, but only at potentials negative to the diastolic potential range. Similar to previous studies conducted under comparable recording conditions [29], [33], I_f_ from AVN cells under control conditions here was small or absent over the diastolic potential range and the lack of any statistically significant effect of ET-1 at potentials positive to −100 mV makes it unlikely that modulation of I_f_ by ET-1 contributed significantly to effects seen under AP recording conditions. By contrast, the activation of an inwardly rectifying K^+^ current, with significant ET-1 activated outward current between ∼−40 and −80 mV is of central importance to the overall actions of ET-1 seen here. The ability of ET-1 to activate a current with similar properties to the muscarinic potassium current (I_K,ACh_) was first demonstrated for non-pacemaker cardiac cells [54], [55], although some studies have demonstrated an ability of ET-1 to inhibit I_K,ACh_ and I_K,Ado_ (e.g. [56]–[58]). Tanaka and colleagues demonstrated activation by ET-1 in rabbit SAN cells of an inwardly rectifying K^+^ current similar to that seen here, also showing this to be Ba^2+^-sensitive and to carry outward current over potentials relevant to the diastolic potential range [21]. Drici and colleagues have demonstrated that ACh-induced AV block in guinea-pig hearts is prevented by tertiapin at concentrations including that used here, without non-selective effects of tertiapin on other important cardiac ionic currents [47]. Thus, the inhibition by tertiapin-Q of the ET-1 activated (Ba^2+^-sensitive) inwardly rectifying current in AVN cells seen here identifies the underlying channels as similar to those that mediate I_K,ACh_. It is also notable that activation of a similar K^+^ current (I_K,Ado_) contributes significantly to the known negative chronotropic and dromotropic effects of adenosine [30], [59]. Moreover, it is evident from the complete lack of membrane potential hyperpolarisation when ET-1 was applied to cells treated with tertiapin-Q, that this current mediated the rapid hyperpolarisation and quiescence of AVN cells following ET-1 application. I_K,ACh_ from the SAN has been shown to exhibit a time-dependent fade in the presence of continued muscarinic receptor activation as a result of desensitization [60], [61] and our results indicate that a similar phenomenon occurs for ET-1 activated inwardly rectifying K^+^ current in AVN cells. The mechanism underlying this effect remains to be elucidated and warrants future study. The progressive MDP depolarisation and decrease in AP amplitude seen when ET-1 was applied to tertiapin-Q treated cells can be explained, wholly or in part, by ET-1 inhibition of I_Kr_ (which would predispose towards membrane potential depolarisation) and of I_Ca,L_ (which would decrease AP amplitude and may also offset AP prolongation anticipated with pure I_Kr_ inhibition), neither of which response showed time-dependent fade. Similarly, the persistence of an ET-1 effect following ET-1 washout, namely maintained quiescence and progressive membrane potential depolarisation (Figure 1), can be accounted for by continued ET-1 receptor activation following washout and by the peptide's effects on I_Kr_/I_Ca,L_ predominating as the inwardly rectifying current response became desensitized/faded. Our data in this regard are compatible with previously reported quasi-irreversible binding of ET-1 to cardiac myocytes ET (ET_A_) receptors and on ET-receptor mediated signalling [39].

### A central role for ET_A_ receptors

Autoradiographical examination of the human cardiac conduction system has demonstrated the presence of both ET_A_ and ET_B_ receptors in the AVN and atrio-ventricular conduction system [24], with a higher proportion of ET_B_ receptors and lower proportion of ET_A_ receptors in the AV conduction system than in the surrounding interventricular and interatrial septa [24]. mRNA for both ET receptor types has been demonstrated to be widespread in the rabbit heart [23], with mRNA levels for ET_B_ in the SAN suggested to be similar to those in atria and ventricles, whilst those for ET_A_ have suggested to be lower [23]. Against this background, it is notable that effects of ET-1 on SAN action potentials and ionic currents have been reported to be highly sensitive to ET_A_ receptor blockade [21], [23]. Inhibitory effects of ET-1 on SAN I_Ca,L_ and I_K_ and the activation of I_K,ACh_-like inward rectifier current were all abolished by selective ET_A_ blockade [21]. Our data provide strong evidence for a similar pivotal role for ET_A_ receptor involvement in ET-1 modulation of AVN cell activity, both at the level of effects on spontaneous APs and also for ionic currents. It is particularly notable that application of the ET_B_-selective agonist IRL-1620 failed to replicate the rapid membrane potential hyperpolarisation and quiescence produced by ET-1 (and inhibited by BQ-123); whilst subsequent ET-1 application in the maintained presence of IRL-1620 led rapidly to these effects. This indicates that the dominant, rapid-onset effect of ET-1 under our conditions was activation of tertiapin-Q sensitive I_K,ACh_-like current, mediated through ET_A_ receptors. Our data do not preclude entirely potential roles for ET_B_ receptor activation: application of IRL-1620 itself led to a modest reduction in AP magnitude and, under voltage-clamp, of I_Ca,L_ (data not shown). Whether or not these actions of IRL-1620 are mediated through ET_B_ receptor activation *per se* or represent a non-selective action of this agent is unclear at this time. Efforts to pursue this line further through the use of a selective ET_B_ receptor antagonist, RES-701, were confounded by potential contaminating effects that led us to abandon its use. Nevertheless, what is very clear from our experiments is the dominant role played by ET_A_ receptor activation in the observed effects of ET-1, which is in good agreement with prior work on the SAN.

### Limitations, future work and conclusions

Understanding of the cellular electrophysiology of the AVN and its modulation has tended to lag behind that of other cardiac regions, to a significant extent likely due to challenges inherent in isolating and working with single AVN cells. Accordingly, the present study is the first to characterise major actions of ET-1 on AVN APs and ionic currents and has identified a number of marked effects. However, whilst our voltage-clamp data can account for the principal effects of ET-1 seen on spontaneous AVN APs, this does not mean that the basis for all modulatory effects have yet been identified. For example, the underlying basis for the small amplitude spontaneous membrane potential oscillations in quiescent AVN cells following ET-1 treatment (Figure 1) remains to be elucidated. Recent data indicate that AVN activity is influenced by cellular Ca^2+^ cycling, likely through the interaction between Ca^2+^ released from internal stores and the sarcolemmal Na-Ca exchanger (NCX) [10], [62], [63]. ET-1 has been reported to stimulate NCX activity [64], [65] and the effects of ET-1 on AVN NCX remain to be investigated. Additionally, it is well established that in atrial myocytes ET-receptor activation of IP_3_ receptors can influence Ca^2+^ mobilization (e.g [66]–[68]) and IP_3_ receptor activity has recently been proposed to modulate murine SAN activity [69]. Thus, potential additional effects of ET-1 on AVN activity mediated by the modulation of Ca^2+^ handling merits future enquiry. The experimental concentration of ET-1 used in this study (10 nM) lies within the range used in previous rabbit SAN and ventricular cardiomyocyte studies (1–100 nM; e.g. [21], [23], [36]) and is similar to that (8 nM) used to study ET-1 effects on undiseased human ventricular myocytes [52]. It should be noted that, whilst normal plasma levels of ET-1 are low (in the fM-pM range; e.g. [70], [71]), considerably higher pericardial and tissue levels can be measured (e.g. [71], [72]). We are not aware of data on endogenous ET-1 levels in AVN tissue *per se*, but a level of ∼1.2 nM has been measured in rabbit left ventricle [71], whilst in human atrial tissue a mean level of ∼19 nM (range ∼2–64 nM) has been measured [72]. Thus, whilst the ET-1 concentration used in this (and other) studies is likely to exceed circulating plasma levels, it lies within measured cardiac tissue levels [71], [72]. Additional avenues of future investigation opened by the present report include the determination of effects of wide-ranging ET-1 concentrations (cf [21]), the study of intracellular pathways mediating major identified effects, and investigation of effects of ET-1 on autonomic agonist modulation of AVN activity.

To summarise and conclude: the present study demonstrates for the first time that ET-1 activation of ET_A_ receptors produces a rapid suppression of spontaneous activity of rabbit AVN cells. ET-1 also inhibits both I_Kr_ and I_Ca,L_ from AVN cells whilst activating K^+^ current through channels identical to those responsible for muscarinic inwardly rectifying K^+^ current. This current mediates the initial rapid hyperpolarisation and quiescence produced by ET-1, whilst I_Kr_ and I_Ca,L_ suppression are likely to contribute to maintained suppression of excitability. Bolus injection of ET-1 in previous experimental studies on anaesthetised animals [26], [27] is likely to have produced a rapid, local rise in ET-1 in blood perfusing the AVN. Rapid membrane potential hyperpolarisation through activation of an I_K,ACh_-like current and suppression of currents important to AVN AP genesis can account for the AV block subsequently observed in those studies [26], [27] - a proposition that can be tested further through future targeted experiments using intact AVN preparations.
